# Adopting population-based interventions towards sustaining child health services in the midst of COVID-19 in sub-Saharan Africa: application of the socio-ecological model

**DOI:** 10.11604/pamj.2022.41.70.31396

**Published:** 2022-01-25

**Authors:** Hubert Amu, Millicent Edem Adjei, Robert Kokou Dowou, Luchuo Engelbert Bain

**Affiliations:** 1 Department of Population and Behavioural Sciences, School of Public Health, University of Health and Allied Sciences, Hohoe, Ghana,; 2 Have Health Centre, Ghana Health Service, Have, Ghana,; 3 Department of Epidemiology and Biostatistics, School of Public Health, University of Health and Allied Sciences, Hohoe, Ghana,; 4 Lincoln International Institute for Rural Health (LIIRH), College of Social Science, University of Lincoln, Lincoln, United Kingdom

**Keywords:** Child health services, COVID-19, socio-ecological model, sub-Saharan Africa

## Abstract

Child health services remain one of the most cost-effective strategies in reducing child mortality which is still disturbingly high in sub-Saharan Africa (SSA). Efforts by governments and other stakeholders in response to the COVID-19 pandemic have inadvertently disrupted the provision of other essential health services including those focusing on children. This comes at the backdrop of the World Health Organization´s guidelines for countries to sustain priority services while fighting the COVID-19 pandemic. Underpinned by the Socio-Ecological Model (SEM), we propose population-based interventions which could help in sustaining child health services in the midst of COVID-19 in SSA. At the intrapersonal and interpersonal levels, educating mothers during routine community outreach services, during child welfare clinics, and in church/mosques could be useful. Education and sensitization of male partners could also be an important intervention. At the institutional and community levels, we recommend the allocation of more funds to other essential health services including child health services. The training and deployment of more general nurses, community health nurses/officers, and public health officers is imperative. The provision and adherence to COVID-19 preventive protocols at health facilities are also recommended at these levels. At the public policy level, insurance and tax relief packages for frontline professionals providing child health services and micro-credit facilities at reduced interest rates for women could be implemented towards sustaining the utilisation of child health services.

## Commentary

The Novel Coronavirus caused by SARS-CoV-2 (COVID-19) was declared a pandemic by the World Health Organization (WHO) on 11^th^ March 2020 [1]. The virus which was first reported in China in December 2019, quickly spread to all countries and territories around the globe. Like other sub-regions of the world, Sub-Saharan Africa (SSA) has not been spared the devastating effects of the pandemic as the number of new cases keep rising. Countries across the sub-region have responded urgently through the implementation of different management and mitigation measures such as contact tracing, testing, case management, travel and movement restriction, social gathering prohibition, generalized or partial lockdown [2]. The effects of the pandemic are felt almost in all sectors and more profoundly in the health system. One aspect of the health system which has been heavily affected is utilisation of child health services [3]. It is, thus, imperative to ascertain the impact of COVID-19 pandemic on child health services in SSA. This paper is underpinned by the socio-ecological model (SEM) [4]. The model entails nested circles that put the individual most affected by a health issue in the centre surrounded by interpersonal, institutional, community, and public policy constructs. The SEM has been adopted in this commentary to present a population-based perspective on how child health services could be sustained amidst the COVID-19 pandemic in SSA. Table 1 summarises our suggested interventions at the various levels of the SEM.

**Table 1:** interventions for sustaining child health services in the midst of COVID-19

Level	Interventions	
	General	Specific
Intrapersonal and Interpersonal	Promoting positive COVID-19 related attitudes and perceptions while addressing rumors.	Educating mothers during routine community outreach services, during child welfare clinics, and in church/mosques.
	Involvement of male partners in child health services	Education and sensitization of male partners
Institutional and Community	Increasing funding and human resource capacity of health institutions	Allocating more funds to child health services Training and deployment of more general nurses, community health nurses/officers, and public health officers
	Reassurance of women on their COVID-19 safety at the health facilities	Provision and adherence to COVID-19 preventive protocols
Public Policy	Policies on motivation of health professionals	Insurance and tax relief packages for frontline professionals providing child health services
	Economic relief policies for women	Micro-credit facilities at reduced interest rates

**Individual and Interpersonal levels**: these levels of the SEM comprise the individual and those they have direct contacts with such as family, close friends, neighbourhood, work, school, church, and neighbourhood [4]. With the rapid spread of COVID-19, mothers in most SSA countries, for the fear of contracting the virus at health facilities by themselves or their children, avoid taking their children to the health facility to seek child health services including weighing and immunization [5]. Other challenges include the refusal of male partners to allow the women access care for their children at health facilities for fear of contracting the virus at the health facility and spreading it among family members. Strategies such as promoting positive COVID-19 related attitudes and perceptions while addressing rumors could help improve the utilisation of child health services. Specific interventions in achieving this could include education of mothers during routine outreach services and during child welfare clinics. Others include engaging religious to ensure that COVID-19 related education is provided in churches and mosques. The involvement of male partners in child health services during the pandemic could help improve service utilisation in the sub-region. Specific approaches in achieving this could include education and sensitization of the male partners on the need to allow their female partners access child health services with their children or even accompany them to access care.

**Institutional and community levels**: the institutional and community levels of the SEM include societal, religious, and cultural values and influences [4]. These levels of the model relate to various child health services that are delivered in communities. According to the WHO COVID-19 guidelines of 2020, health systems of countries are to continue providing essential services including child health services while combating the COVID-19 pandemic. These child health services are more important during this pandemic to prevent the re-emergence of child killer diseases that were on the verge of eradication in SSA. Even before the pandemic, the financial capacity of the health system in SSA depends marginally on the foreign donors and international donor organizations, however, with the pandemic which is also having a devastating toll on the developed countries, the financial support to SSA is no longer forthcoming [2]. As result, the available meagre financial resources of health facilities that are to use the funding of child health services have been diverted towards the management of COVID-19 in terms of purchasing personal protective equipment (PPE), ventilators and other necessary supplies. The health system of SSA is also faced with a shortage of human resources [6]. The pandemic has exposed this lack of health professionals more pronounced as many of the health professionals are redirected from their previously assigned portfolios in the provision of child health services. Mothers visiting Child Welfare Clinics (CWC) for services may no longer receive the full package of services. For example, children may be immunized but not weighed, thereby receiving an incomplete service package which puts them at risk of not being monitored for preventable health risks including malnutrition. This comes at the backdrop of the United Nations Children´s Fund´s (UNICEF) call on governments and partners to sustain life-saving child health services. This is because, interruptions in immunization services for instance, could result in a resurgence of childhood diseases for which vaccines already exist.

While the primary effects of the pandemic on children appear to be limited, children are highly susceptible to the indirect secondary effects of the pandemic. The preventive measures such as lockdowns, social distancing, the conversion of facilities into COVID-19 treatment and isolation centres, diversion of limited resources for COVID-19 management, reassignment of health professionals for the management of the pandemic have led to a reduction in the utilisation of basic child health services such as immunization [6]. The pandemic has also let to the redirection of funds by governments, donors, and stakeholders towards COVID-19 containment efforts, from other important health services including child health services. This has led to their children being less able to access important health care services. To remedy this situation, the health facilities should take measures to maintain essential services by allocating adequate funds for essential health care including child health services. Efforts should be made by SSA governments to train more health professionals and deploy them to the health facilities to support the existing health professionals. Specifically, there should be training and deployment of more general nurses, community health nurses/officers and public health officers who provide routine community-based outreach child health services. Besides, women should be reassured of their safety in the health facilities when they visit through the provisions and adherence to the necessary COVID-19 precautionary measures including provision of hand washing materials, sanitizers, and face masks.

Public policy level: this level of the SEM includes the influence of public policy on child health services utilisation amidst the COVID-19 pandemic. Policy strategies implemented by nations globally to tackle COVID-19 upon its emergence were purposed at providing adequate care for all patients while minimizing the effects of the pandemic on other health services including child health services [7]. Governments in SSA in response to the pandemic have also enacted policies focusing on testing, restriction, humanitarian supports to the population, health professionals and the financial relief [8]. The policy response plan for COVID-19 in SSA is also targeted at slowing and stopping the transmission, preventing community outbreaks, and effectively managing COVID-19 cases. While these strategies have largely helped in slowing the spread of the virus and prevent the overwhelming of the health facilities, they have inadvertently disrupted other essential services such as child health services. Therefore, it is imperative for the nations in SSA to make policy efforts at sustaining child health services while continuing the fight against COVID-19. An important policy intervention implemented by some governments in SSA in response to COVID-19 has been the provision of motivation packages for health professionals. In Ghana, for instance, frontline health professionals were given special insurance and tax relief packages. The frontline health workers were defined as staff working in testing, isolation and treatment centres, and those who carry out contact tracing [9]. This was done to encourage health professionals in continuing to provide care to the COVID-19 cases and other management services of the pandemic. To sustain child health services, similar policies could be implemented to motivate health professionals who provide such services at health facilities or as routine outreach services in communities. The economic impact of the COVID-19 pandemic on businesses due to the partial or total lockdown as preventive measures and loss of the source of income by many families in the SSA sent many individual households into poverty [10]. Despite the fact the economic impact of the COVID-19 is felt by everyone, women are bearing more of the economic and social fallout as they are more represented in many of the industries and businesses that are hardest hit by COVID-19, such as food and retail services which are small-scale in nature. This economic challenge makes it difficult for many families to seek child health services for their children as they are unable to get money for transportation to access child health services or purchase any related medications. Economic relief policies by governments in the sub-region for such women could help improve child health service utilisation. These could be in the form of micro-credits at reduced interest rates to revamp their small-scale businesses.

## Conclusion

Sustaining child health services is very crucial for SSA countries in reducing child mortality levels to as low as 25 per 1,000 live births by the year 2030. While SSA countries are putting all essential measures in place to fight COVID-19, effort should also be made to sustain child health services utilisation. At the intrapersonal and interpersonal levels, educating mothers during routine community outreach services, during child welfare clinics, and in church/mosques could be useful. Education and sensitization of male partners could also be an important intervention. At the institutional and community levels, we recommend the allocation of more funds to other essential health services including child health services. The training and deployment of more general nurses, community health nurses/officers, and public health officers is an imperative. The provision and adherence to COVID-19 preventive protocols at health facilities are also recommended at these levels. At the public policy level, insurance and tax relief packages for frontline professionals providing child health services and micro-credit facilities at reduced interest rates for women could be implemented towards sustaining the utilisation of child health services.
